# Plasma Surface Modification of the Inner Wall of Montgomery’s Tracheal Implant (T-Tube)

**DOI:** 10.3390/polym16223223

**Published:** 2024-11-20

**Authors:** Konstantin G. Kostov, Ananias A. Barbosa, Fellype do Nascimento, Paulo F. G. Cardoso, Ana C. P. L. Almeida, Antje Quade, Daniel Legendre, Luiz R. O. Hein, Diego M. Silva, Cristiane Y. Koga-Ito

**Affiliations:** 1 Laboratory of Plasma and Applications, Department of Physics, School of Engineering and Sciences, São Paulo State University (UNESP), Guaratinguetá 12516-410, SP, Brazil; ananias.alves@unesp.br (A.A.B.); fellype@gmail.com (F.d.N.); ana.c.almeida@unesp.br (A.C.P.L.A.); 2 Thoracic Surgery Research Laboratory (LIM61), Division of Thoracic Surgery, Instituto do Coração, Hospital das Clínicas HCFMUSP, Faculdade de Medicina, Universidade de São Paulo, São Paulo 01246-903, SP, Brazil; cardosop@gmail.com; 3 Leibniz Institute for Plasma Science & Technology—INP, 17489 Greifswald, Germany; quade@inp-greifswald.de; 4 Adib Jatene Foundation—FAJ, São Paulo 04014-002, SP, Brazil; daniel@fajbio.com.br; 5 Laboratory of Materials Images, Department of Material and Technology, School of Engineering and Sciences, São Paulo State University (UNESP), Guaratinguetá 12516-410, SP, Brazil; rogerio.hein@unesp.br; 6 São José dos Campos Institute of Science and Technology (ICT), São Paulo State University (UNESP), São José dos Campos 12245-000, SP, Brazil; diego.m.silva@unesp.br (D.M.S.); cristiane.koga-ito@unesp.br (C.Y.K.-I.)

**Keywords:** cold atmospheric plasma, plasma jet transfer, tracheal implant, surface modification

## Abstract

Tracheal stenosis (i.e., the abnormal narrowing of the trachea) can occur due to a variety of inflammatory and infectious processes as well as due to therapeutic procedures undertaken by the patient. The most common cause of tracheal obstruction in patients has been prolonged intubation. Depending on the extent of the stenosis and its exact location, the surgical insertion of a tracheal stent is the only option for addressing this issue. The Montgomery T-tube implant is a valuable tracheal stent made from medical-grade silicone that provides a functional airway while supporting the tracheal mucosa. However, its performance is subject to gradual deterioration due to biofilm colonization of the stent’s inner wall, which may explain the discomfort claimed by many patients and clinical failures. Recently, cold atmospheric plasmas (CAPs) have emerged as an alternative technology to many conventional medical procedures, such as wound healing, skin treatment, decontamination of medical devices, etc. Here, we report on plasma-induced surface modification of the inner wall of a T-tube implant, considering future biomedical applications. To generate the plasma, we employed a cold atmospheric pressure plasma jet in gas helium, which was directly inserted into the T-tube implant. To assess the treatment uniformity, the degree of surface modification and its extension along the stent’s inner wall was analyzed using different process parameters.

## 1. Introduction

**Figure 1:**
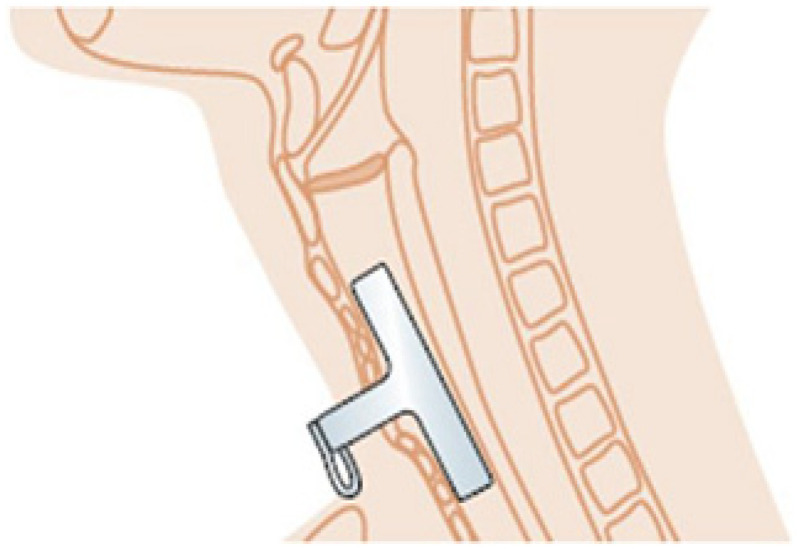
Illustration of a typical T-tube stent implanted in a patient.

Tracheal stenosis is a serious medical condition characterized by the narrowing of the trachea, which can lead to significant breathing difficulties. This airway constriction can occur for various reasons, such as trauma, tumors, inflammatory diseases, and post-intubation injury [1,2]. Since many COVID-19 patients remained intubated for a long period of time, a substantial increase in the number of instances of iatrogenic tracheal stenosis can be expected. Patients with tracheal stenosis may experience shortness of breath, wheezing, difficulty swallowing, frequent respiratory infections, etc. [2]. Treatment for tracheal stenosis varies depending on the patient’s condition and underlying cause, but in the most severe cases, it includes surgical intervention and the insertion of a stent, which is placed in the narrowed section of the trachea to hold it open [1]. Among different types of medical devices, silicone stents have drawn special attention because they are well tolerated, easy to adjust for a specific patient, and relatively inexpensive [3]. They are typically made of medical-grade silicone, which is biocompatible and flexible, reducing the risk of tissue irritation. Montgomery’s tracheal implant, the T-tube, is a specialized silicone stent designed for patients with tracheal and laryngeal stenosis or other airway disorders [2,3,4]. The stent is inserted in the patient’s throat via bronchoscopy under full anesthesia and remains there to provide a secure airway [3]. The implant is shaped like the letter “T”, with a long vertical segment placed within the trachea and a shorter horizontal segment extending outside the throat (see Figure 1). In a medical emergency, this opening provides air access to the patient’s lungs and can be used to administer drugs.

Tracheal stents can significantly improve breathing, but not without potential complications.

For instance, a significant challenge with the use of any long-term indwelling medical device, such as the T-tube, is biofilm formation. Microbial colonization is one of the reasons for device replacement in up to 70% of cases [5]. Biofilms are structured communities of bacteria and other microorganisms that adhere to surfaces and produce a complex extracellular matrix. The process begins with the initial attachment of bacteria to the tube surface, followed by colonization and biofilm maturation, eventually leading to the release of planktonic bacteria that can spread infections. A persistent biofilm inside the T-tube can cause chronic inflammation of the surrounding tissue, leading to discomfort, increased mucus production, and further airway narrowing. Biofilms are highly resistant to antibiotics and the host immune response, making infections difficult to treat. The management of biofilm-related complications requires periodical replacement of the T-tube by an endoscopic procedure under general anesthesia. The stent changes typically vary between 6 months to over 1 year, depending on the patient’s health status and habits [3]. Therefore, the control of biofilm formation may reduce its local interaction with the diseased tracheal mucosa and may allow safer and lengthier time of use of T-tube implants. Such factors can potentially reduce the risk for the patient and medical costs. Since the biofilms are resistant to antibiotics and cannot be removed from the implant’s inner wall by scrubbing or rinsing, an alternative approach such as the cold plasma application [6] should be employed.

Plasma is an ionized gas consisting of ions, electrons, neutrals, free radicals, and excited particles that emit ultraviolet light. Depending on their temperature, plasmas are classified into two large groups—thermal plasmas and non-thermal or cold plasmas. Unlike thermal plasmas, which are very hot, cold plasmas can operate at low temperatures, making them suitable for applications involving delicate samples and biological tissues without causing thermal damage [7]. Cold plasmas can be generated in certain conditions, even in an open environment and at atmospheric pressure, thus eliminating the need for a vacuum chamber [8]. In the last two decades, cold atmospheric pressure plasma has emerged as a groundbreaking tool for the surface modification of materials [9]. The plasma surface interaction can include etching, surface activation, and cross-linking [10]. Plasma etching involves creating micro-scale roughness on the surface that enhances adhesion properties. Plasma can also induce the cross-linking of polymer chains, enhancing the surface’s mechanical strength and chemical resistance. Surface cleaning and activation are other effects of plasma-surface interaction. Reactive species in the plasma can introduce new functional groups (e.g., hydroxyl, carboxyl) to the surface, increasing its surface energy and improving its wettability [10]. Since the plasma generated in the air contains plenty of reactive oxygen and nitrogen species as well as energetic photons, it is highly effective in sterilizing medical instruments and surfaces. In comparison to chemical disinfectants, cold plasma normally produces none or very little amounts of harmful residues, ensuring a safer environment for patients and healthcare workers [11]. The plasma’s ability to inactivate many pathogens, including antibiotic-resistant bacteria, makes it a valuable tool in preventing hospital-acquired infections [12]. Moreover, plasma can inactivate cancer cells and stimulate tissue regeneration. These unique features of non-thermal plasmas open up many possibilities in medical treatments, sterilization, and biological research [13,14,15]. Some works [16,17,18] have especially shown that cold plasma, in general, prevented biofilm formation on different medical devices.

Another interesting plasma feature is that a tiny plasma jet can be produced or injected inside narrow tubing and small cavities so that plasma reactive species interact with the device’s inner wall to improve biocompatibility and reduce microbial colonization [18]. However, generating plasma inside long tubes is still challenging [19]. For instance, in [20], a 1-m-long, 4-mm-diameter silicone tube was sterilized by low-pressure plasma, and the process effectiveness was compared to one of the conventional sterilization agents, such as UV radiation and ozone. A 60-cm-long silicone tubing with a diameter of 4 mm was successfully sterilized by using the gas products generated by a plasma torch operating at atmospheric pressure [21]. The latter was produced close to the silicone tube end, and the plasma-induced reactive species were carried along the tube by a gas flow. S. Jin et al. [22] proposed a sterilization method that relies on a helium discharge ignited inside a tube by a sequence of external ring electrodes. This approach works for long tubes, but it requires too many ring electrodes. Finally, in [23], T. Wong et al. demonstrated surface modification of the inner wall of a 30-cm-long, 2 mm inner diameter PTFE tube by a small He plasma jet, which was introduced inside the plastic tube. This specially designed semi-flexible plasma jet has a length of 2.5 m, and it was gradually inserted in the PTTE tube until it reached its opposite end. The authors demonstrated that the plasma jet enhanced the material-wetting properties in a uniform manner. However, depending on the size of the PTFE tube, the process required complex manipulation of the plasma jet (insertion plus a simultaneous rotation). Another recent paper [24] described a plasma jet device for treating upper respiratory tract infections. In this work, an in vitro safety assessment showed that the cold plasma jet can eliminate microbes under established conditions without any signs of cell damage. A comprehensive literature review of in vitro and in vivo experiments that utilized cold atmospheric plasmas on implants, as well as a discussion about plasma antimicrobial efficacy, was presented in a recent article [25].

As previously mentioned, the T-tube is a valuable tracheal implant; however, its performance is subject to gradual deterioration due to biofilm colonization of the stent’s inner wall. On the other hand, as reported in [20,21,22,23], tiny plasma jets can be produced or introduced inside tubes and cavities to decontaminate the device’s inner surface. Therefore, one can speculate that plasma jet application inside a T-tube implanted into a patient may reduce microbial adhesion to the stent inner wall, as well as inactivate some microbes already presented there. For instance, the in vitro study [26] demonstrated that a plasma jet, generated inside a T-tube, could effectively reduce the microbial load. At the same time, the viability of human cells isolated from normal bronchial epithelium and exposed to plasma was not affected by the treatment. Therefore, one can expect that such a process is safe for patients, and when the plasma treatment is performed periodically inside the T-tube stent, it will greatly reduce the rate of biofilm formation, thus increasing the implant’s time of use.

Although several previous works [19,20,21,22,23,24,25] have demonstrated that plasma processing of the inner wall of plastic tubes and catheters is possible, there has been no investigation on plasma treatment of the inner wall of tracheal implants so far. At first glance, such a process should not be a problem because the Montgomery stents typically have a 9–10 mm inner diameter, allowing easy plasma jet manipulation. However, a challenge in treating the stent’s inner wall would be its 3D geometry, which differs from the straight catheters and plastic tubes reported previously. Moreover, the proposed process, the feasibility of which is the subject of this work, is supposed to be performed in a T-tube already implanted inside a patient. Our idea is to conduct the treatment by introducing a cold plasma jet through the short sleeve of the T-implant (the one that terminates outside the patient’s throat). This way, the plasma will only interact with the stent’s inner wall without direct contact with the patient’s body. However, the reactive species generated by plasma are carried by a gas flow and spread inside the stent, affecting microbial cells and eventually mitigating the biofilm formation. In this way, the treatment of a T-tube implanted in a patient could be, in principle, performed periodically in an ambulatory environment without the need for anesthetic or surgical intervention. Since plasma reactive species have a strong bactericidal effect, one can expect that the biofilm growth rate will be greatly reduced, resulting in a longer time of use of the T-tube.

The aim of this study is to provide a proof of principle that the cold atmospheric plasma jet produced by a device reported previously [27] can mitigate the biofilm formation inside the T-tube. For this purpose, a cold plasma jet inserted directly through its shorter section treated the inner wall of a pristine T-tube stent (without biological matter). In this preliminary study, we addressed issues crucial to determining the process feasibility, such as: (1) Is a cold plasma jet introduced inside the stent capable of modifying the implant’s inner wall? (2) What is the degree of surface modification and extension along the implant’s long sleeve? (3) Is the plasma modification effect uniform? If the answers to those questions are satisfactory, the door for future in vitro and in vivo studies of the mitigation of biofilm formation inside T-tube implants will be opened. Eventually, this investigation may result in a new therapeutic procedure based on cold plasma application to enhance the lifetime of T-tube implants inside patients.

## 2. Experimental Setup, Materials, and Methods

### 2.1. Plasma Source

**Figure 2:**
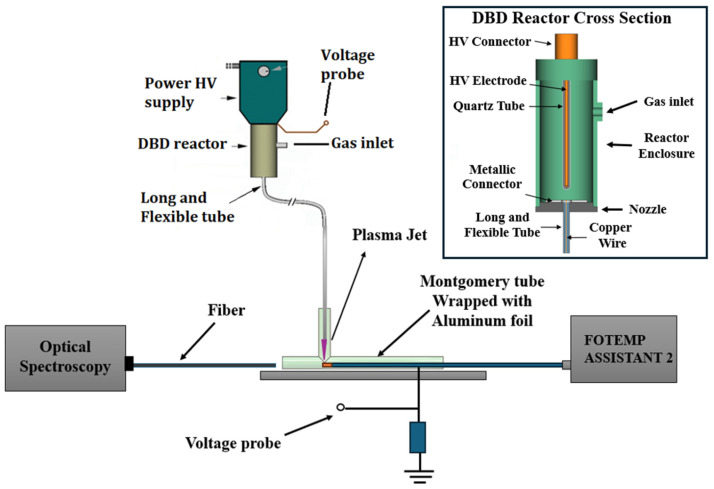
Experimental arrangement and reactor description.

For this study, we employed the homemade device previously reported [27]. This device can generate a cold plasma jet at the end of a 1.0-m-long plastic tube, thus allowing precise and controlled delivery to a targeted area. Figure 2 depicts a schematic layout of the experimental setup.

The device consists of an encapsulated pin electrode centered in a cylindrical dielectric barrier discharge (DBD) reactor. The reactor’s exit nozzle is connected to a 3.3-mm-diameter polypropylene (PP) tube. Along the tube passes a thin (Ø = 0.25 mm) copper wire at a floating potential, which terminates a few mm before the tube’s orifice. The working gas, He (99.2% purity, from Air Liquide Brazil, São José dos Campos, Brazil), is injected in the DBD chamber at a flow rate of 2.0 slm. From the reactor, the gas propagates along the tube and is ejected in the ambient through the tube’s far end. When the discharge in the primary DBD reactor is on the wire tip, it ignites a small plasma jet, which is expelled through the distal end of the flexible plastic tube. The tube material is chosen to withstand the conditions created by the plasma, such as heat, high voltage, and potential reactive species, and it can be held by hand without the risk of electric shock. The tube’s flexibility allows maneuverability, enabling the plasma to reach difficult or confined spaces, making it a versatile tool for various applications [18]. The plasma system is driven by a commercial pulse voltage generator fabricated by Ibramed Ltd.a, Amparo, SP, Brazil, designed for dermatological applications. It generates an adjustable sequence of dumped HV oscillations (1–5) repeated with the line frequency (60 Hz). More details about the device operation, its parameters, the generated reactive species, and the gas temperature can be found in [27]. Importantly, all plasma jet characteristics, such as the patient leaking current, UV radiation, ozone production, etc., are within the safety limits established for medical applications when using He as the working gas [28].

Based on previous experiments carried out by our research groups, we chose a gas flow rate of 2.0 slm (standard liters per minute), because such a value was the one that presented the best results in inactivating microorganisms with less cellular toxicity and little damage to living tissues [26,27]. He was chosen as the working gas due to the good antimicrobial results already obtained and also due to the lower electric current values that have been obtained with it when compared to argon gas. The electric current values are essential for patient safety in in vivo treatments; this is why we chose He gas rather than Ar.

**Figure 3:**
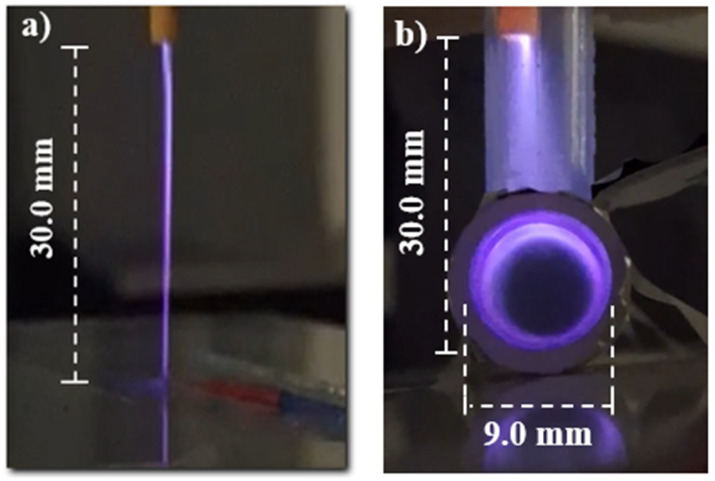
Photographs of (**a**) He plasma jet in an open environment impinging on a dielectric surface, and (**b**) the plasma produced inside the T-tube.

Figure 3 shows two photos of the plasma jet operating in different environments: Figure 3a exhibits the plasma jet launched in open space, while the plasma generated inside the T-tube can be seen in Figure 3b. In our experiments, the plasma jet was inserted into the shorter T-tube segment, which was kept in a vertical position. The tip of the plastic tube, from where the plasma jet is ejected, can be seen at the top of Figure 3b. As can be seen, the plasma jet expands downward and eventually spreads into the horizontal segment of the T-tube.

### 2.2. Montgomery T-Tube Stent

Tracheal implants are crucial for ensuring adequate airflow in patients, and the size of a stent is vital for its effectiveness. Selecting a tracheal stent’s appropriate size involves carefully considering the patient’s unique anatomy, the extent of tracheal narrowing, and the underlying condition. For instance, the stent should be long enough to cover the entire area of stenosis without extending into the bronchi. If the soft silicone stent is too long for a certain patient, the surgeon can easily adjust it simply by cutting the tube’s excess length. The stent’s diameter should be slightly larger than the tracheal lumen to prevent migration but not so large as to cause excessive pressure on the tracheal walls. Therefore, conforming to their outer diameter, the T-tubes are divided into three categories: small (5–10 mm), medium (10–14 mm), and large (14–20 mm) stents. The small ones are typically used in pediatric patients or adults with smaller tracheal lumens, while the large stents are used in cases of significant tracheal obstruction or large patients with bigger tracheal diameters. The medium-sized diameter stents (12 mm) are the most common and are normally implanted in adult patients with moderate tracheal stenosis or other airway-compromising conditions. In this work, we used only standard, 12-mm-diameter (OD), polydimethylsiloxane (PDMS) stents provided by the Adib Jatene Foundation, São Paulo, Brazil. The PDMS used for this T-tube is a high-consistency elastomer Class VI obtained by platinum-cure chemistry. It is a medical-grade silicone polymer designed for healthcare applications, which complies with the biocompatibility requirement of ISO 10993 [29]. The manufacturer also produces stents with 8-, 10-, and 14-mm outer diameters.

### 2.3. Samples Preparation

**Figure 4:**
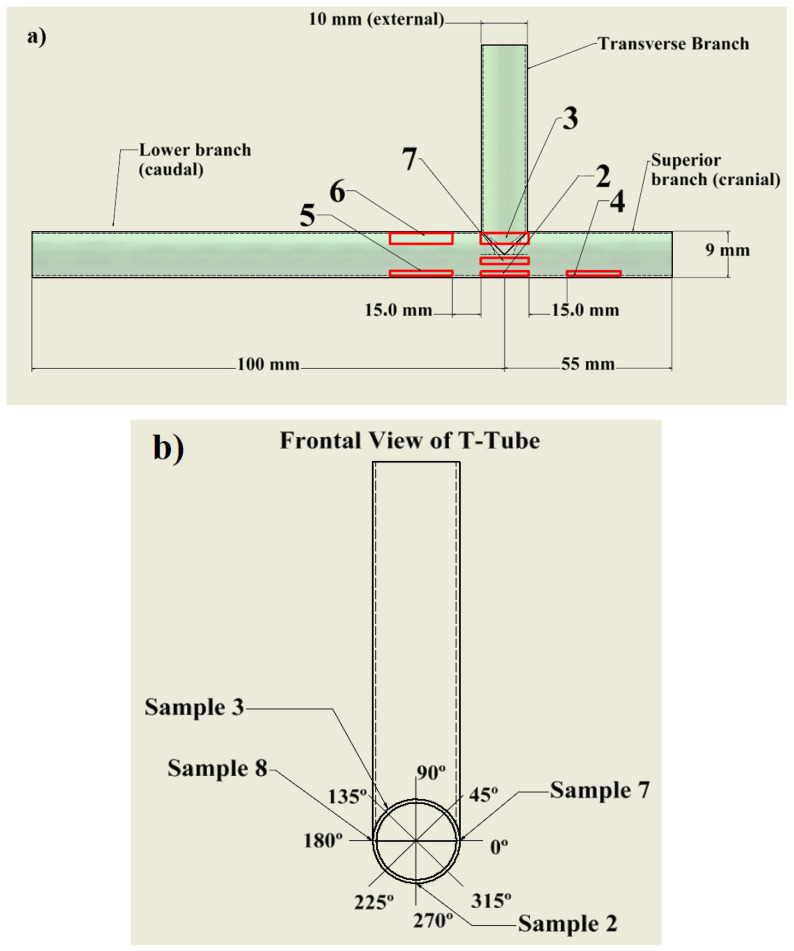
Schematic drawings showing (**a**) a lateral and (**b**) a frontal view of the T-tube with its main dimensions and the exact stent’s locations from where samples were cut for further analysis.

The T-tube was plasma-treated as received, i.e., without any previous pretreatment or cleaning. Figure 4 shows drawings of the employed silicone stent with its exact dimensions. Plasma was injected into the standard T-tube through its shorter branch (see Figure 3b). In preliminary tests, we employed the same plasma jet to treat biofilms formed on PDMS discs with a diameter of 8 mm, and different treatment times were also evaluated. Good results for biofilm inactivation were obtained starting from 5 min of plasma exposure [26]. Then, based on this and on the more complex geometry and larger dimensions of the T-tube, we decided that the most appropriate duration for the plasma treatment would be 10 min. After the plasma processing, the silicone stent was carefully cut into small pieces (approximately 15 mm × 5 mm) that were further analyzed to assess the surface chemistry and morphology. Figure 4a depicts the different stent regions from where samples for X-ray Photoelectron Spectroscopy (XPS) Axis Supra, Kratos, UK, GB and Scanning Electron Microscopy (SEM) analysis were extracted. In this study, we focused mostly on the region close to the implant’s T-junction for several reasons. First, the total length of a pristine T-tube is quite large (155 mm), so the plasma treatment effect at the tube’s edges will be minimal. Secondly, when a T-tube is implanted, depending on the patient’s physical characteristics, its length is reduced preferentially from the caudal branch. Finally, medical studies have shown that the biofilm forms predominantly at the region around the stent’s T-junction.

### 2.4. Surface Characterization Techniques

#### 2.4.1. Wettability

Most polymers have low surface energy and consequently exhibit hydrophobic characteristics. Enhancement of material wettability by plasma is a well-established technique. Moreover, the wettability can be easily assessed by contact angle measurements (CAs), which is a cheap and simple method. The wettability of the T-tube inner wall was assessed by a Ramé-Hart 300 F1 goniometer (Succasunna, NJ, USA). For this purpose, samples with the approximate length of 15 mm, cut from different positions at the T-tube, were analyzed. For each sample, three drops of deionized water (1.0 µL) were deposited along the bottom line of the curved surface. DropImage software version 2.10.04 (Oslo, Norway), provided with a goniometer can only automatically determine the water contact angle (WCA) on a flat surface. Therefore, in this case, we only used the equipment CCD camera to take pictures of the droplets. Then, these pictures were processed one by one with ImageJ software 1.54g, Java 1.8.0_345 (64-bit) (NIH, Bethesda, MD, USA), which has a special applicator for drawing tangent lines to droplets sitting on a curved surface and can give the contact angles on the droplet’s left and right sides. The WCA values reported in this paper are the mean values from the right-side and left-side WCAs measured at three different spots on each sample. This procedure for WCA determination should be repeated manually for all droplet images, and thus it is time-consuming; the obtained standard deviation is around 4 degrees. Because of the hydrophobic recovery of plasma-treated polymers (aging effect), all samples for WCA measurements were analyzed within 1 h after the plasma exposure.

#### 2.4.2. XPS

The surface elemental composition of the silicone implant’s inner wall was examined using X-ray photoelectron spectroscopy (XPS) (Kratos AXIS Supra instrument, Manchester, UK). Three kinds of XPS measurements were performed: a wide scan (one measurement at the center of each sample) with 150 W X-ray radiation (15 kV, 10 mA) and 160 eV pass energy. Secondly, element scans (line scans at 1 mm) with 150 W X-ray radiation (15 kV, 10 mA) and 80 eV were performed along the longest sample dimension (parallel to the T-tube axis). Then, the distribution of the sample’s elemental composition was analyzed to determine the homogeneity of the treatment. XPS data provided insights into the uniformity and effectiveness of the treatment across the entire T-tube inner surface. Finally, the highly resolved C 1s spectra were measured at the center of the samples. The binding energy of the main component in C 1s was set to 284.7 eV and was used to calibrate all spectra. N 1s was only detected in traces with a fraction of <0.2 at%, close to the detection limit. The XPS data were processed with CasaXPS 2.3.15 software.

#### 2.4.3. SEM

Possible alterations of the surface morphology of the T-tube inner wall after the plasma treatment were evaluated by scanning electron microscopy (SEM) using a Carl Zeiss microscope model EVO LS-15. To avoid surface charging during the SEM analysis, the samples were covered with a thin (few nm) golden film. The analysis of SEM images was performed using Gwyddion (http://gwyddion.net/), a modular, cross-platform, open-source software licensed under the GNU General Public License. The Gwyddion software provides a tool for calculating the root mean square surface roughness (*R_q_*) of multiple rows and columns of a given image. The *R_q_* value is calculated as:

(1) $$R_{q}(RMS)=\sqrt{N^{-1}\sum_{i=1}^{N}{\left(z_{i}-z_{m}\right)}^{2}}$$

where *N* is the number of data points in the area under analysis, *z_i_* is the height of the i-th peak, and *z_m_* is the average peak height of the area.

#### 2.4.4. Gas Temperature Inside the Stent

To measure the gas temperature inside the T-tube that was reached during the plasma treatment, we employed a fiber optic temperature (FOT) sensor from Weidmann, Dresden, Germany. The data acquisition of the gas temperature profile was performed in two different modes. First, temperature measurements were carried out along the horizontal T-tube branch by displacing the FOT sensor for a fixed plasma jet outlet position d of 35 mm inside the T-tube vertical branch. Secondly, the FOT sensor position was fixed right below the plasma jet outlet, and the temperature measurements were performed as a function of *d*; i.e., changing the position of the plasma jet inside the vertical branch of the stent.

#### 2.4.5. Parameters of the Treatment’s Electrical and Optical Characterization

Before the treatment, a Montgomery implant was placed on an isolated horizontal platform with its short segment pointing vertically upward. The implant’s shaft was wrapped with a thin Al foil and grounded through a serial resistor of 100 Ω. The electrical characterizations of the device consisted mainly of measuring the applied voltage $v\left(t\right)$ via a P6015A Tektronix HV (1000:1) probe and the discharge current i(t). The latter was obtained by measuring the voltage across a serial 100 Ω resistor. The electrical signals were recorded on a Tektronix 3032C oscilloscope (Beaverton, OR, USA), and the discharge power *P_dis_* was then calculated using the relation (1), where *f* = 60 Hz is the repetition rate of the voltage signal, with *T* = 1/*f*.

(2) $$P_{dis}=f\int_{0}^{T}v\left(t\right)\cdot i\left(t\right)\;dt$$

To identify the excited species produced by the plasma jet inside the T-tube, optical emission spectroscopy (OES) in the UV-visible range of 200–750 nm was performed using an Avantes spectrometer model AvaSpec-ULS2048X64T (Apeldoorn, The Netherlands), which had a spectral resolution (FWHM) equal to 0.76 nm. The light was collected with an optical fiber that was positioned on the axis of the horizontal branch of the T-tube.

## 3. Results and Discussions

**Figure 5:**
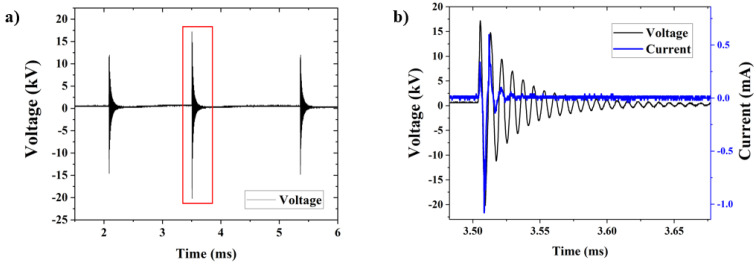
Typical current and voltage signals: (**a**) a waveform of the applied voltage signal showing three consecutive packages of damped sinewave oscillations and (**b**) current and voltage waveforms recorded in a single burst of HV oscillations (indicated in a red frame in (**a**)).

Figure 5a depicts the typical waveform of the applied voltage signal. It consists of three consecutive high-voltage bursts with a magnitude of up to 20 kV_peak_. The repetition rate of the whole set of three pulses is 60 Hz. The current and voltage signals within the second HV burst are shown in Figure 5b.

**Figure 6:**
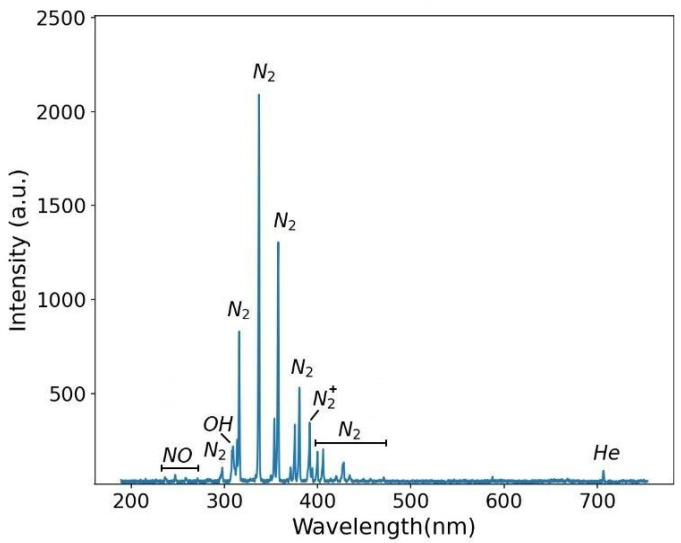
The optical emission spectrum exhibits the excited species produced by the plasma jet inside the T-tube.

To modify the T-tube’s inner wall, the tip of the plastic tube was inserted into the vertical sleeve of the stent down to a certain depth *d*, which could be adjusted. In most experiments, however, the plasma outlet was kept 25 mm inside the stent, i.e., about 30 mm above the bottom of the inner stent wall. For these operating conditions, the input discharge power *P_dis_* was ≈1.4 W. In all experiments, the He gas flow rate was fixed at 2.0 slm, and the time of plasma application inside the stent was 10 min. A typical emission spectrum of the plasma inside the T-tube is presented in Figure 6. As can be seen, the spectrum is dominated by the emission bands of excited nitrogen molecules (N_2_, second positive system) in the wavelength range 297 nm–467 nm. A molecular nitrogen ion (N_2_^+^) band emission was also observed at 391 nm. The emission band from the OH radicals was detected at 309 nm. Emissions from the NO radical were observed with very low intensity between 240 nm and 260 nm. These excited species were formed by the interaction between the He plasma jet and the air molecules trapped inside the T-tube. Very weak atomic emission from He was also detected at 706.5 nm.

After the plasma treatment, the stent was cut into pieces, and the inner wall characteristics were evaluated by SEM, XPS, and wettability analysis.

### 3.1. Morphological Modifications

**Figure 7:**
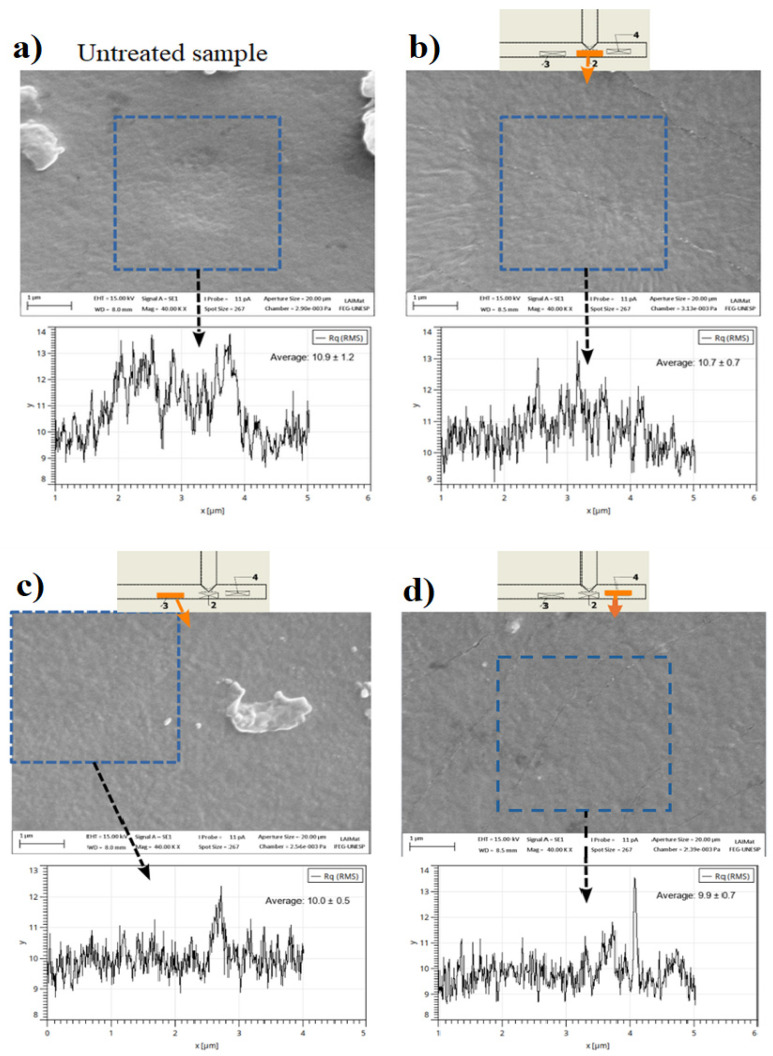
SEM images showing: (**a**) the surface of a pristine implant and (**b**–**d**) the T-tube inner surface after the plasma exposure. The images in (**b**–**d**) were taken from samples extracted from different positions on the T-tube (indicated above the picture).

Figure 7 depicts different micrographs of the implant’s inner surface and the average 1-D profiles calculated in the 4 µm × 4 µm regions highlighted in the images. Following the manufacturer’s specifications, the T-tube was made from a polydimethylsiloxane (PDMS) composite, which, in addition to being optically transparent, having a low cost and high capability to replicate models, also exhibits good thermal and mechanical performance [30]. As shown in Figure 7a, this material exhibits a very smooth surface with some dust particles on it (the T-tube was treated as received, i.e., without previous cleaning). After the treatment (see Figure 7b–d), the implant’s surface visibly does not change. However, a detailed image analysis performed in several different sample locations indicates some slight differences. For example, the large protruding on the control sample that was partially removed by the plasma, as can be seen when comparing the average profiles in Figure 7, as well as the lower relative uncertainty in the average *R_q_* values. Those differences suggest a mild etching on the plasma-treated samples, which agrees with results from previous studies where PDMS samples were exposed to APPJ treatment [31,32].

### 3.2. Surface Elemental Composition and O Content Distribution

**Figure 8:**
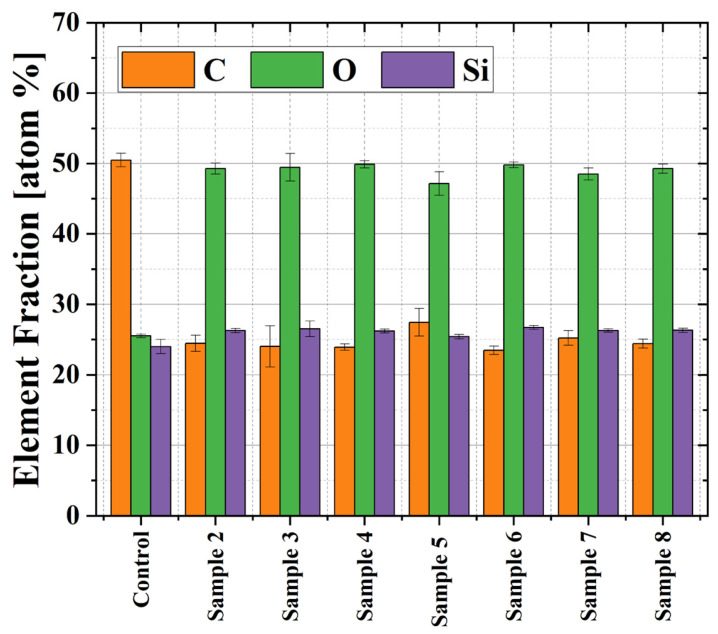
Elemental compositions of the untreated sample 1 (control) and of the samples exposed to plasma treatment (#2–8).

The wide scans of pristine and plasma-treated samples can be found as complementary material to this article. They reveal that the plasma exposure led to a decrease in the C 1s peak and an increase of the O 1s peak, while the Si 2p remained unchanged in amplitude. Figure 8 presents the elemental composition of sample 1 (control), which was cut from an untreated T-tube, and the composition of samples 2–8 extracted from different parts of the plasma-treated T-tube (see Figure 4a).

The untreated sample exhibits an elemental composition of 50.5 at% C, 25.5 at% O, and 24.0 at% Si, which is similar to one of the polydimethylsiloxane (PDMS) polymers [32,33]. As reported in [34], low-pressure oxygen plasma treatment of PDMS led to a substantial incorporation of O atoms and a decrease in C concentration, while the silicon content remained almost unchanged. In our case, we obtained a very similar trend—after the plasma treatment, the oxygen content of the T-tube inner wall increased while the carbon percentage decreased by half (see Figures S1 and S2 in the Supplementary Materials). As can be seen independently of the samples’ location after the plasma treatment, the average O content almost doubled. This is quite a good result considering that the samples were extracted from different locations on the Montgomery implant. It means that the gas flow carries the reactive species produced by the plasma jet and can effectively spread inside the entire horizontal branch of the T-tube.

**Figure 9:**
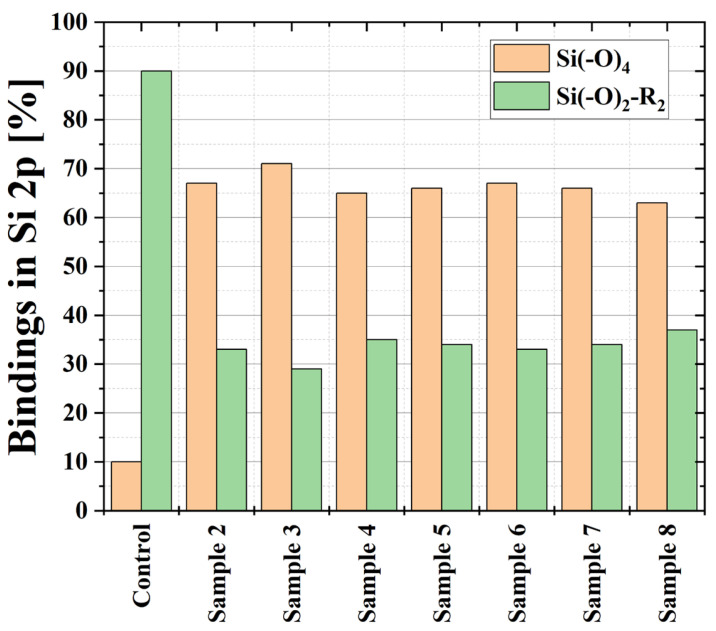
Bindings in Si 2p measured for the untreated sample (control) and for the samples exposed to plasma treatment.

Although the Si content of the treated samples remained virtually unchanged, the Si binding changed as revealed by the shape of the Si 2p peak. It was resolved into two components. The pristine PDMS sample contained mainly Si(-O)2R2 bonds (~90%) at 101.5 eV and only a small amount (~10%) of inorganic Si(-O)4. Details of the Si 2p deconvolution can be seen as Figures S3 and S4 in the Supplementary Materials. After the treatment, the shape of all Si 2p peaks changed, and an increase in the silica-like Si(-O)4-bindingat 103.6 eV was observed. Depending on the position of the samples during the treatment, the content of the Si(-O)4-binding varies from 60% to 70%, as can be seen in Figure 9. This finding suggests that the plasma treatment of the T-tube led to the formation of inorganic silica-like bonds on the surface of PDMS polymer, like the ones observed in [34] after irradiation with UV light.

**Figure 10:**
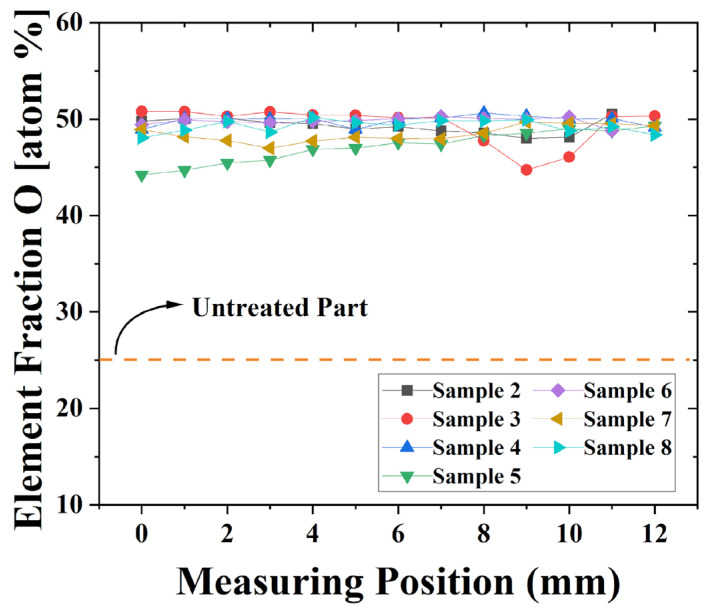
Oxygen content measured along the longer side of the different plasma-treated samples.

A detailed, space-resolved XPS analysis performed along each plasma-treated sample demonstrated rather uniform elemental compositions. The elemental content distributions for all plasma-treated samples can be found as Figure S5 in the Supplementary Materials. To overview these results in just one plot, the O content measured along all samples is presented in Figure 10. The exact sample locations on the treated T-tube are presented in Figure 4. As can be seen, the plasma exposure led to a substantial (twofold) O increase with practically homogeneous longitudinal distribution for almost all samples. The notable exceptions are samples 3 and 4, cut from the lateral (vertical) part of the T-tube wall, the O percentages of which are not uniformly distributed. The O content of sample 3 exhibits a small minimum, which is close to the T-tube junction. The complex gas flow pattern in this region probably caused this finding. On the other hand, for sample 5, the O percentage at the left side (the further sample’s edge) gradually decreases.

**Figure 11:**
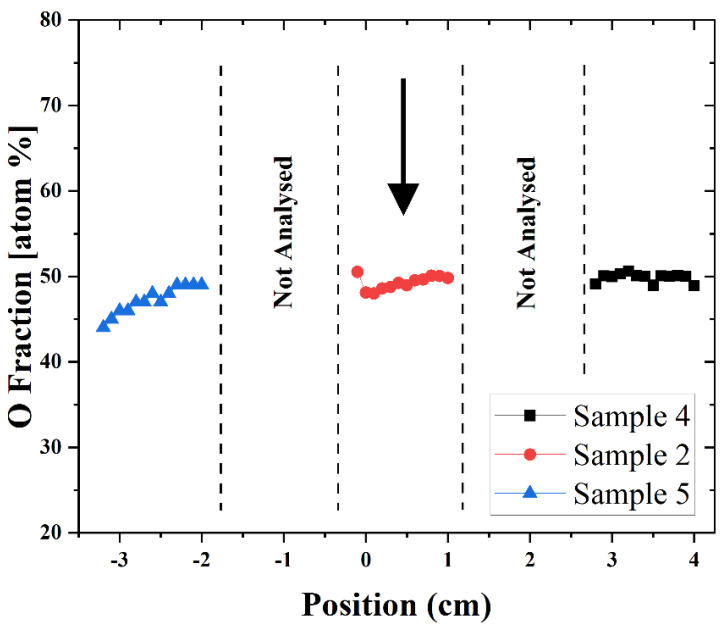
A composite plot presenting the O content along the three samples (5–2–4) that are located on the same axis. The arrow indicates the approximate position of the plasma jet in the T-tube during the treatment.

Since the reactive species have a short lifetime, they can cover only a limited distance inside the stent. Therefore, to estimate the extent of the plasma modification effect along the entire T-tube length, the O content of samples 2, 4, and 5, situated at the same angular position, is plotted against the distance (see Figure 11). This composite plot contains two regions that were not analyzed by XPS. However, the O content there can be roughly extrapolated by using the O values at the neighboring sections. The plasma effect on the T-tube inner wall, indicated by the higher O content of around 50%, spreads over a length of more than 7 cm. The gradual O decrease detected at the left end of sample 5 is caused by the reduced quantity of reactive species that can reach that region. Still, a plasma modification effect, although reduced (O atoms content is around 45%), is presented at that position and probably it extends a little bit further.

### 3.3. Water Contact Angle

**Figure 12:**
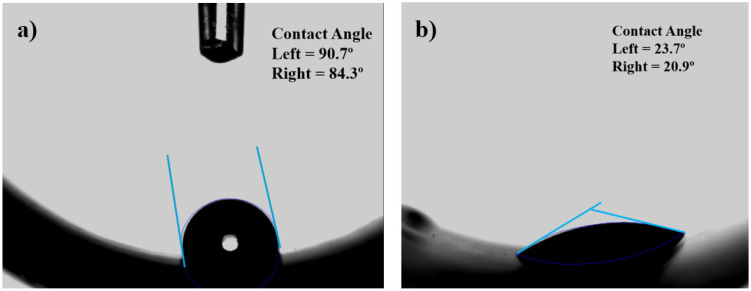
Photos of WCA measured on the concave surface of the T-tube for (**a**) pristine sample and (**b**) a sample treated with plasma.

PDMS is a typical polymeric material with low surface energy that exhibits hydrophobic characteristics [30,35]. Figure 12 shows photographs of water droplets placed on the inner surface of pieces (half-cylinders) cut from a T-tube. The untreated sample in Figure 12a shows a high contact angle of 87.5°. As shown in Figure 12b, plasma treatment led to a significant WCA reduction (22.3°). This behavior is expected, considering that the XPS analysis detected the incorporation of polar O-related groups on the T-tube inner surface after the treatment.

### 3.4. Gas Temperature Inside the T-Tube

**Figure 13:**
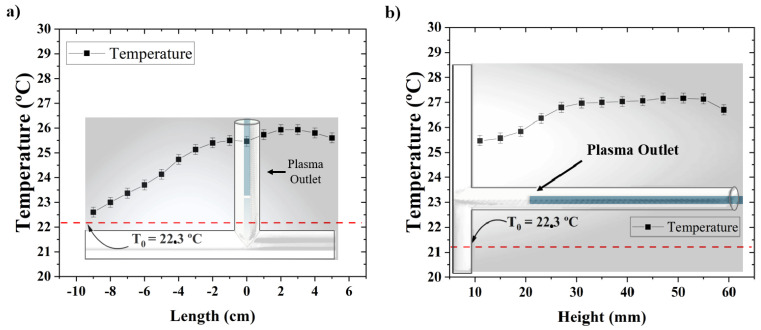
Gas temperature distributions. (**a**) Temperature profile along the T-tube horizontal segment obtained for a fixed position (*d* = 35 mm) of the plasma jet, and (**b**) temperature at the bottom of the T-tube right under the plasma jet measured for different jet positions inside the vertical segment. The red dashed line indicates the ambient temperature at the time of measurement.

Before any in vivo plasma jet application, one should guarantee that the gas temperature is below the limit established for living tissues (40 °C). We measured the gas temperature inside the T-tube under two different conditions. Firstly, at a fixed jet depth (*d* = 35 mm) inside the vertical stent branch, the temperature along the horizontal T-branch was measured by moving the FOT sensor. The temperature profile is presented in Figure 13a. It exhibits a distribution with a plateau-like shape, the highest values of which (about 4 °C above the ambient temperature) are close to a position right under the plasma jet. The maximal gas temperature at these conditions barely exceeds 26 °C, which is significantly below the safety limit. In a second experiment, the FOT sensor was kept fixed at the T-tube junction, while the jet position inside the vertical T-tube branch was varied. The results are shown in Figure 13b. The maximal gas temperature that was reached was under 28 °C; i.e., again well below 40 °C. Therefore, one can conclude that, at this experimental configuration (a cold atmospheric pressure He jet inserted into the shorter T-tube branch), the gas temperature inside the implant is suitable for in vivo applications.

## 4. Conclusions and Future Work

A plasma jet ignited at the end of a flexible plastic tube represents a significant technological advancement, offering precision, versatility, and safety across various applications. The present study aims to provide proof of concept that a cold atmospheric pressure plasma jet applied inside a T-tube implant can modify its inner wall. The plasma jet was introduced through the stent’s transversal branch, which terminates outside the patient’s throat, thus allowing easy access to the inner part of the implant. Therefore, in principle, such plasma application can mitigate biofilm formation on the inner T-tube wall. This investigation shows that plasma treatment greatly enhances the inner wall wettability by incorporating polar O-related groups on the surface. The plasma treatment bindings of Si atoms on the T-tube’s inner wall changed, indicating a formation of an inorganic silica-like layer on the PDMS surface. The modification effect inside the horizontal T-tube branch spans in a relatively uniform manner, covering a distance of at least 7 cm. The finding is important for future applications because it demonstrates the feasibility of this approach. The plasma jet did not significantly alter the surface morphology of the silicone implant. The next step will be an in vitro experiment, where a biofilm previously grown on the inner wall of the horizontal T-tube branch will be exposed to reactive plasma species using the experimental parameters determined in this pilot study.

## Data Availability

The original contributions presented in the study are included in the article, further inquiries can be directed to the corresponding author.

## Supplementary Materials

The following supporting information can be downloaded at: https://www.mdpi.com/article/10.3390/polym16223223/s1, Figure S1: Wide XPS scan of sample #1 (cut from pristine T-tube); Figure S2: Wide XPS scan of sample #3 (cut from plasma-treated T-tube); Figure S3: High-resolution XPS scan of Si2p peak of sample #1 (pristine T-tube); Figure S4: High-resolution XPS scan of Si2p peak of sample #3 (treated T-tube); Figures S5: (a–g) Detailed view of the O content of plasma-treated samples (2–8) measured by XPS. The scan was made along the longitudinal sample dimension with resolution of 1 mm.
