# Supplementary material for: Plasma Surface Modification of the Inner Wall of Montgomery’s Tracheal Implant (T-Tube)
Source: Polymers (Basel). 2024 Nov 20;16(22):3223. doi: 10.3390/polym16223223 (PMC11598400; doi:10.3390/polym16223223)
Supplement: Supplementary file 1 [file polymers-16-03223-s001.zip › polymers-3281006-supplementary.pdf]

Fig. S1. Wide XPS scan of sample #1 (cut from pristine T-tube)

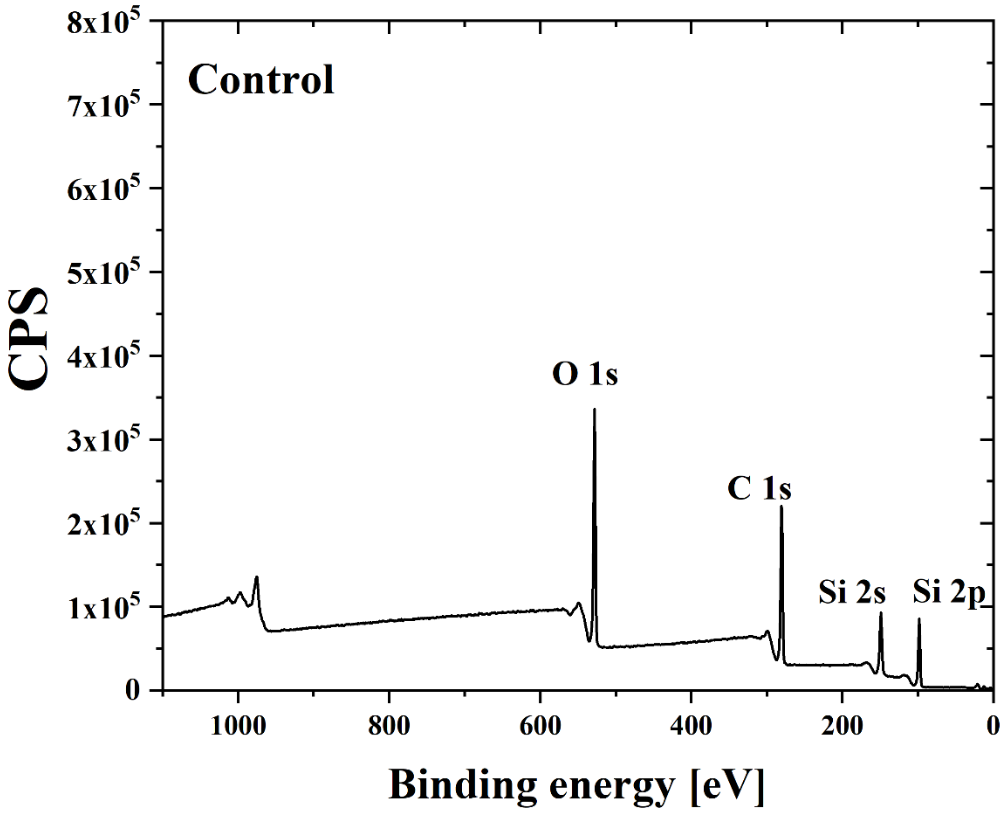

Fig. S2. Wide XPS scan of sample #3 (cut from plasma-treated T-tube)

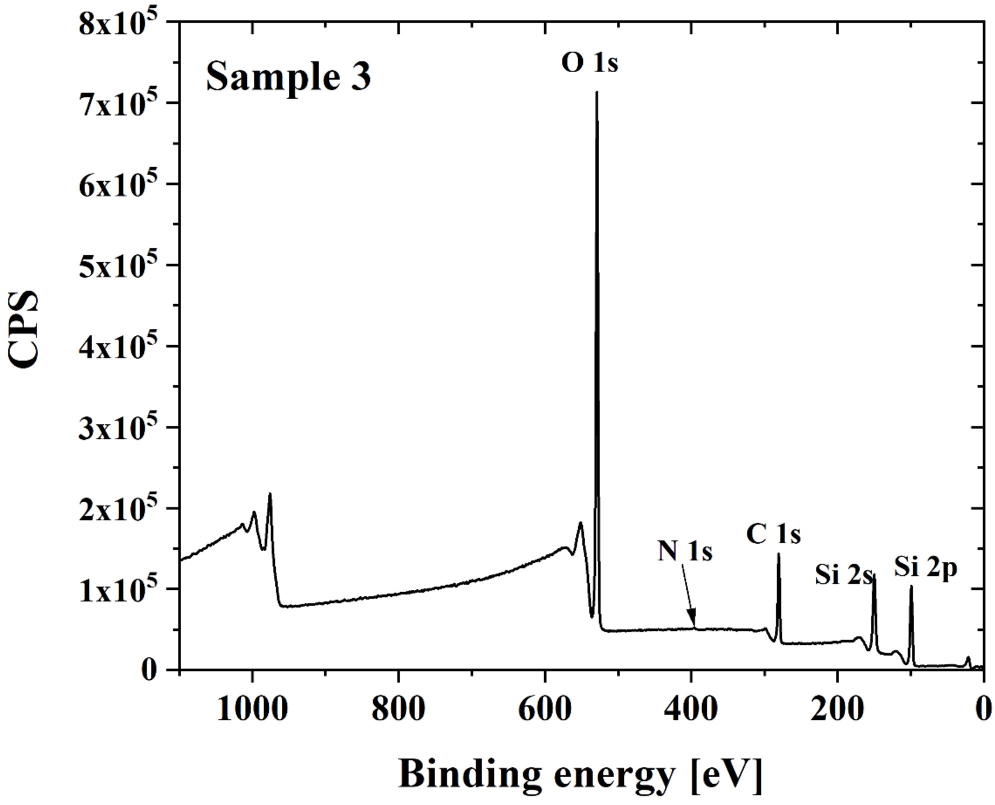

Fig. S3. High-resolution XPS scan of Si2p peak of sample #1 (pristine T-tube)

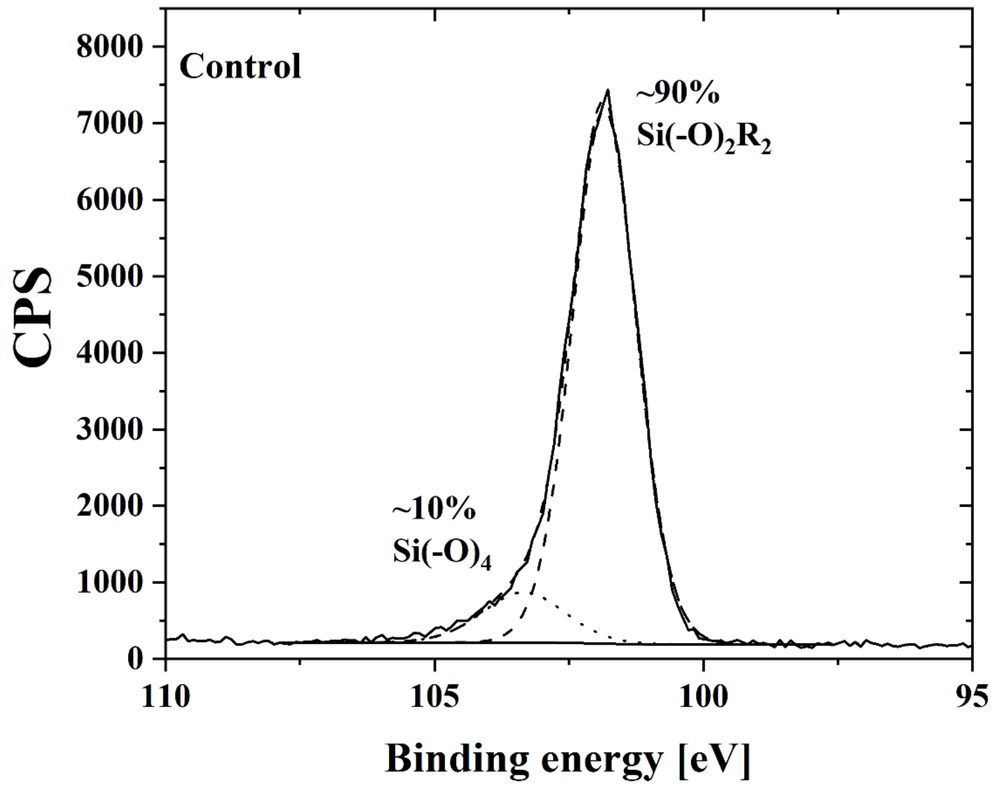

Fig. S4. High-resolution XPS scan of Si2p peak of sample #3 (treated T-tube)

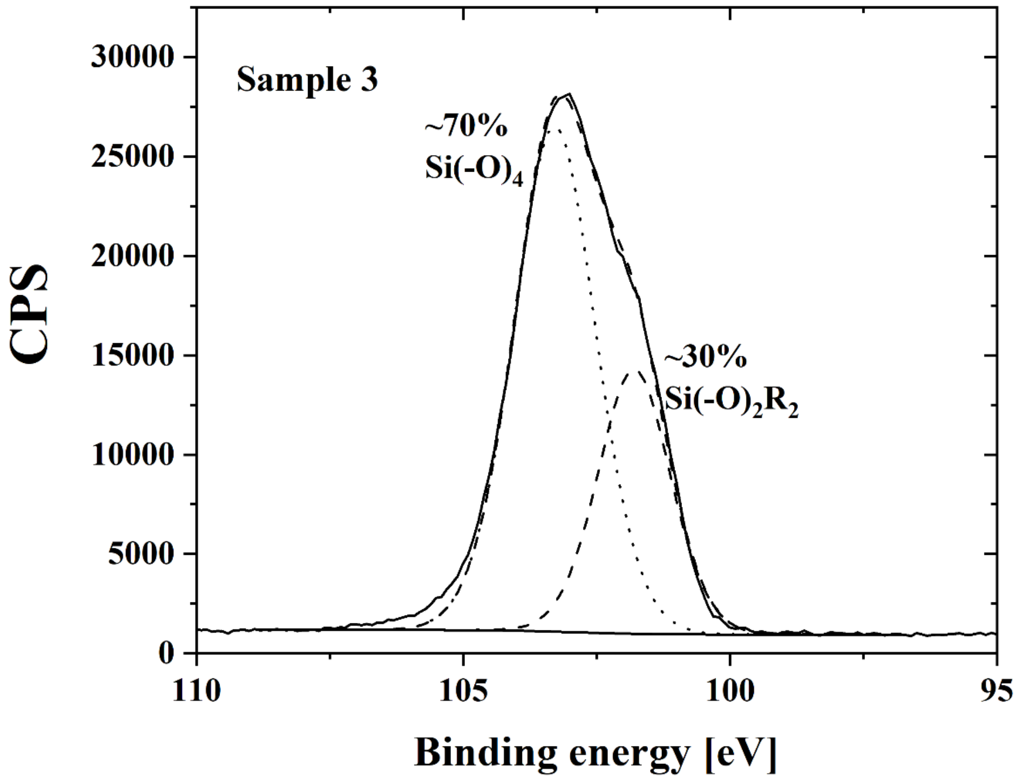

Fig. S5 (a-g). Detailed view of the O content of plasma-treated samples (2-8) measured by XPS. The scan was made along the longitudinal sample dimension with resolution of 1 mm.

(a)

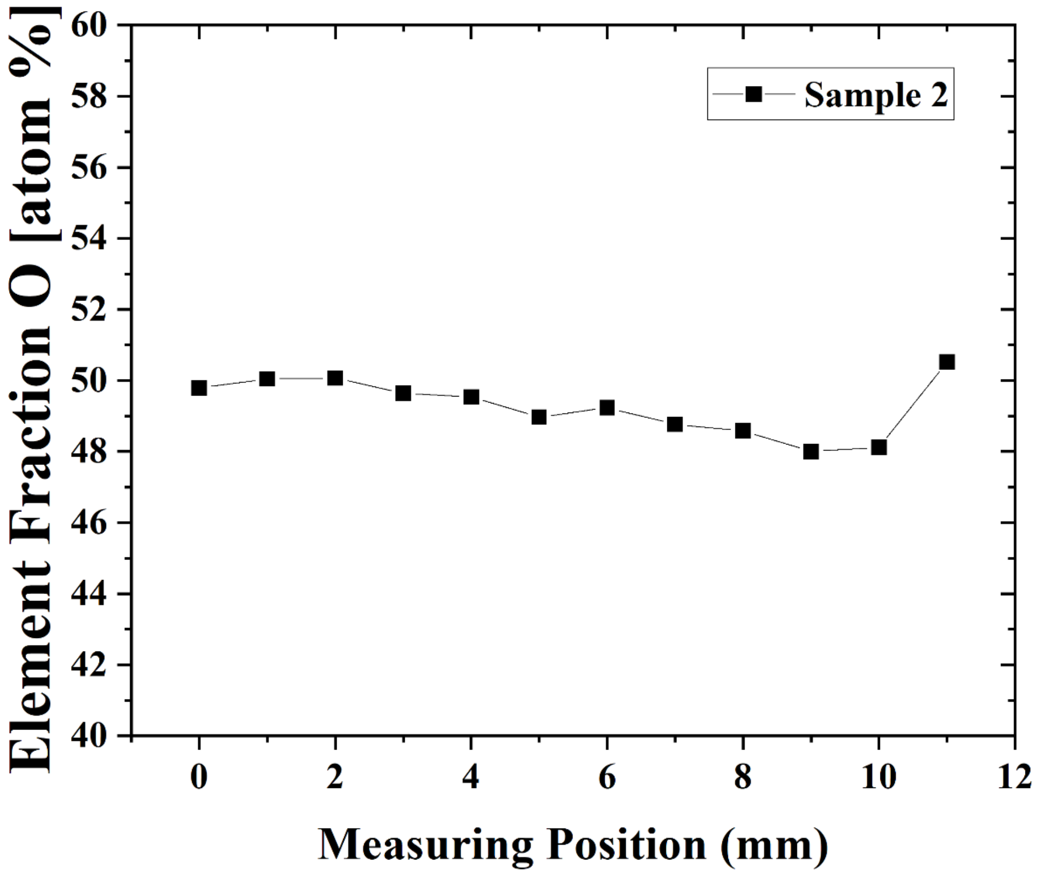

(b)

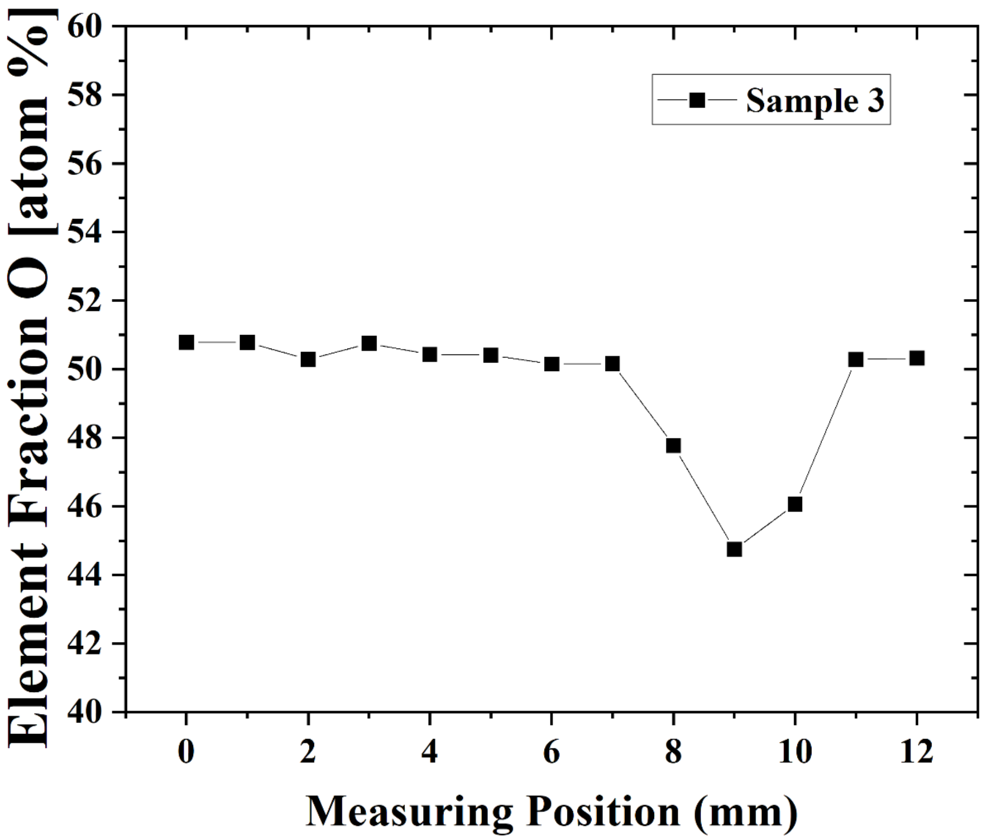

Sample 3
Element Fraction O [atom %]
Measuring Position (mm)
60
58
56
54
52
50
48
46
44
42
40
0
2
4
6
8
10
12

(c)

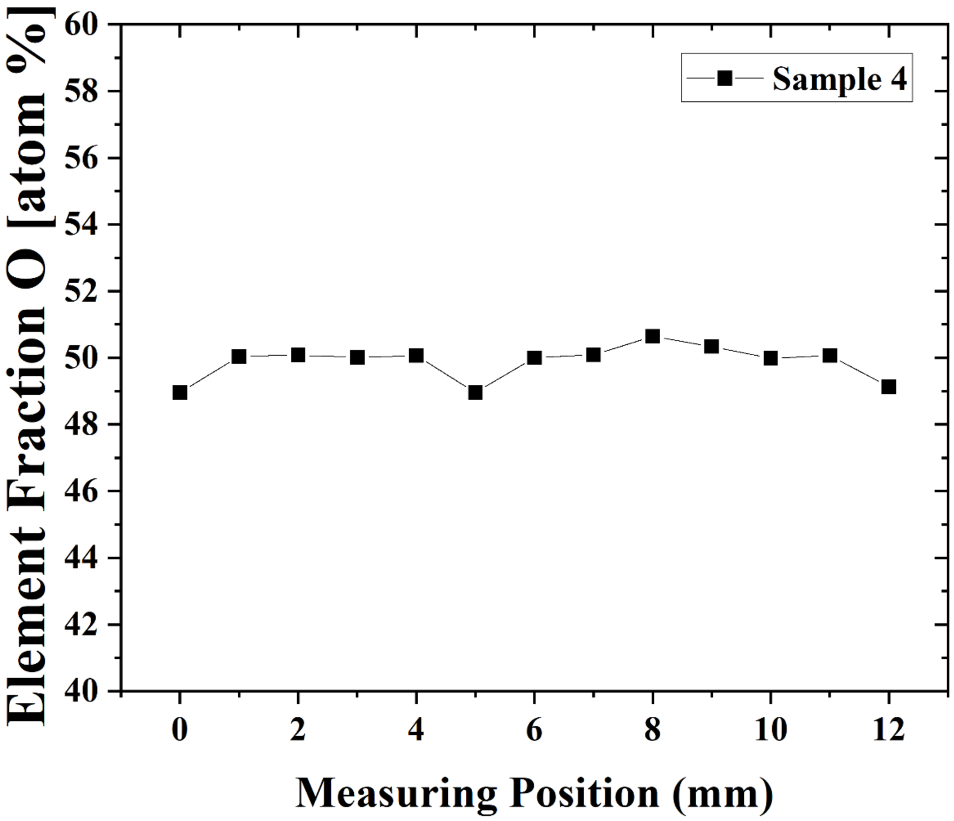

Sample 4
Element Fraction O [atom %]
Measuring Position (mm)
60
58
56
54
52
50
48
46
44
42
40
0
2
4
6
8
10
12

(d)

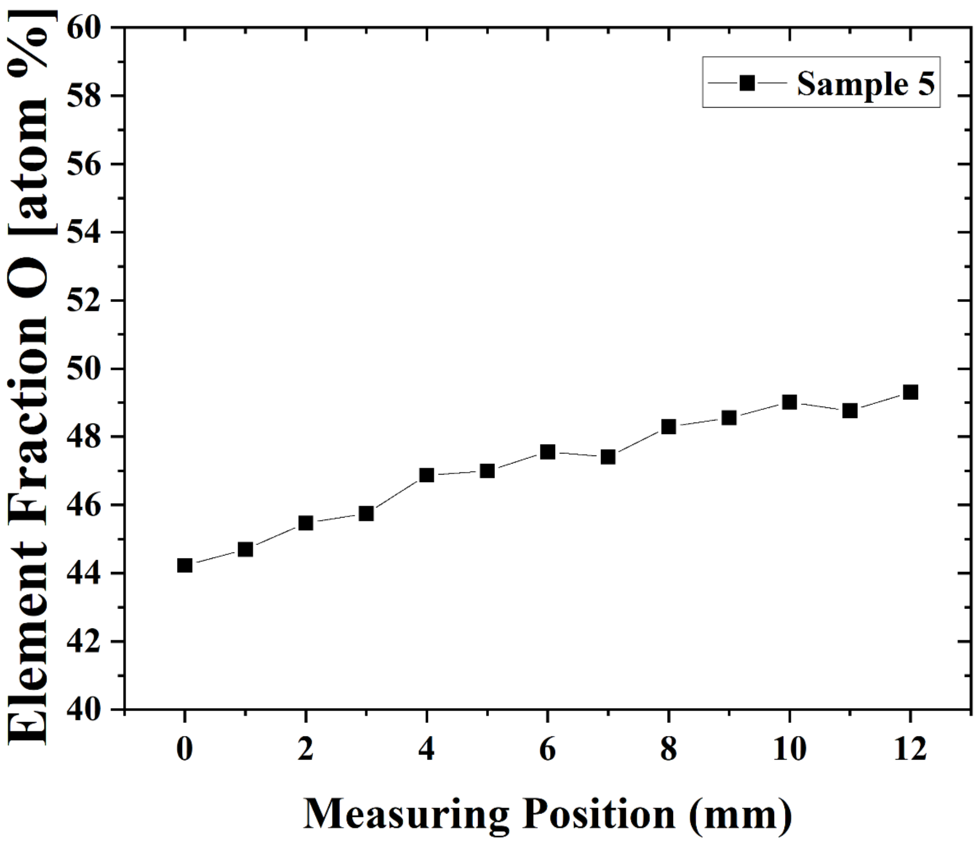
Sample 5
Element Fraction O [atom %]
Measuring Position (mm)

(e)

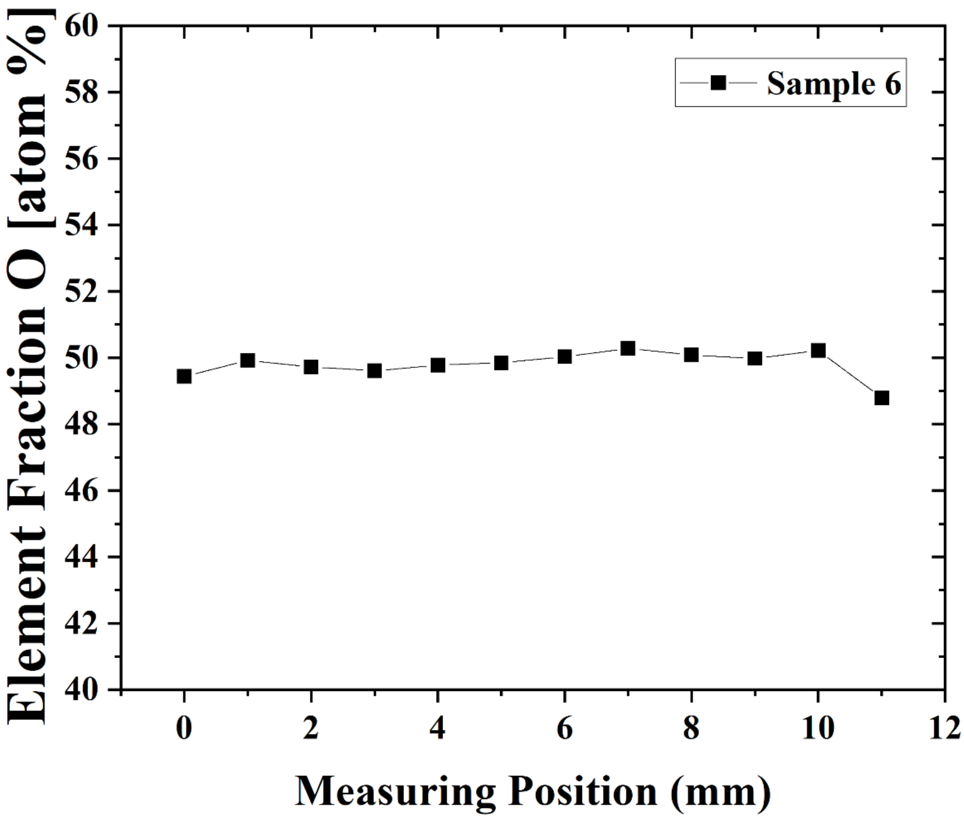
Sample 6
Element Fraction O [atom %]
Measuring Position (mm)

(f)

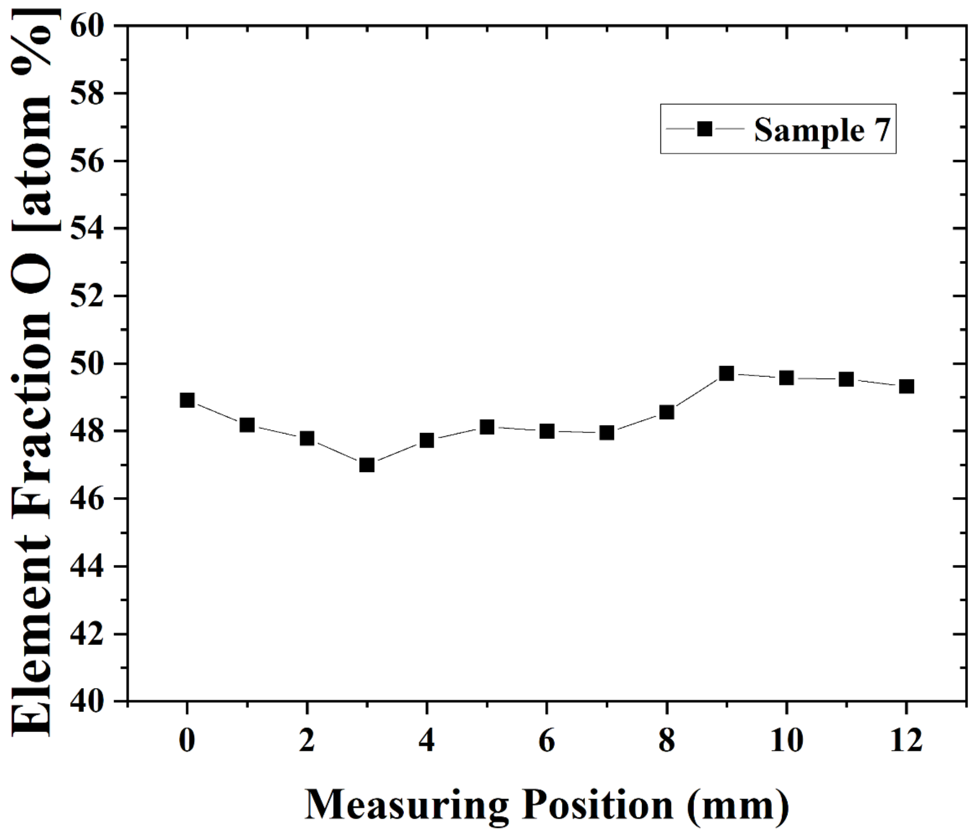
Sample 7
Element Fraction O [atom %]
40
42
44
46
48
50
52
54
56
58
60
0
2
4
6
8
10
12
Measuring Position (mm)

(g)

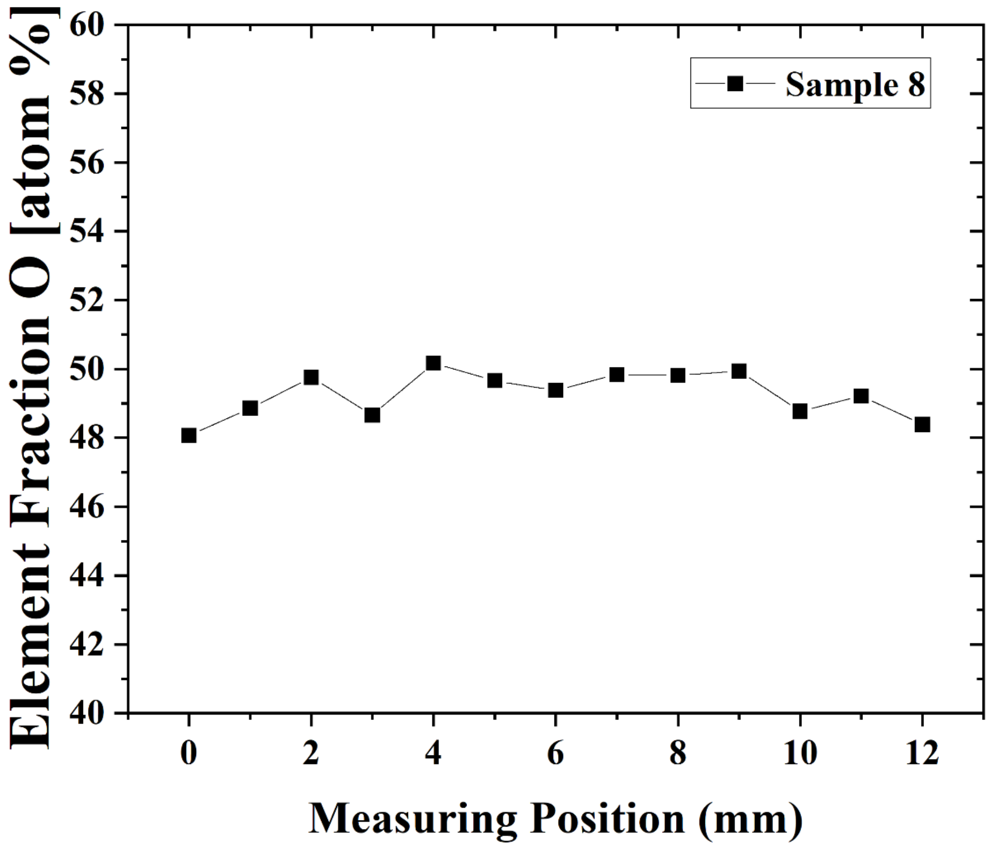
Sample 8
Element Fraction O [atom %]
40
42
44
46
48
50
52
54
56
58
60
0
2
4
6
8
10
12
Measuring Position (mm)
